# 

**Published:** 1889-06

**Authors:** 


### SPECIAL.

We shall send, during the present month, to each subscriber in arrears a bill of his indebtedness, and earnestly request that the amount be remitted to as without further delay. It is needed in carrying forward our work.

### BOOK NOTICES.

BABY LAND AND OUR LITTLE MEN AND WOMEN, D. Lothrop Co., Boston. It is absolute cruelty to little folks to deprive them of these monthly visitors.

A NEW REVELATION of the Revelations of the New Testament, by Prof. H. B. Philbrook, has just been completed and is soon to be brought out, the author informs us. This whole subject has been such a puzzle to us, we shall be glad of any reasonable solution of it. The last discourse we listened to from this subject was by Hon. A. H. Daley who struggled manfully with the "Green Bay Tree," till it spread all over spiritualism.

THE CENTURY MAGAZINE. We have so frequently spoken in praise of the above excellent monthly, that additional words seem almost superfluous. It must be seen and read to be properly appreciated, not only once, but every month. The amount of literary and artistic labor expended upon this periodical is surprising, and every subscriber gets the full benefit of it. Century Company, New York.

ELECTRICITY IN FACIAL BLEMISHES, by Prof. Plym S. Hayes, A. M. M. D. This compact little volume deals not only with the "Histology of the skin and hair," in a brief and comprehensive manner, but also with the causes of those hairy and nævi disfigurements which so often blemish the fairest features: at the same time it furnishes a complete manual of operatic cure by "the employment of electrolysis," which has stood the test of thirteen years of practical application. To the practitioner of surgery and medicine, it will be found of great value. W. J. Kuner, Publisher, 96 Washington st., Chicago.

PSYCHIC SCIENCE, by Hudson Tuttle, New York. This is a most instructive volume of 250 pages. The author has given a great deal of attention and study to his theme, and has for many years been prominently before the public as a writer upon kindred subjects. In his opening chapter he quotes as a sort of forerunner or text, Guizots well-known aphorism "Belief in the supernatural (spiritual) is the special difficulty of our time ; denial of it is the form of all assaults on Christaianity, and acceptance of it lies at the root, not only of Christianity, but of all positive religion whatever."

The book takes a wide range over modern fields of thought, including Christian Science, Mind Cure, Faith Cure, and their Psychic Revelations, fortified with numerous individual instances of the independent manifestation of the spirit, or the action of the spirit temporarily released of its physical environment. Take it all in all, it is a work of great value to the student whose reaches after knowledge extend beyond the material plane, into the higher realms of truth. For sale by M. L. Holbrook & Co., New York City- Price $1.25.

